# Molecular characterisation of fowl adenovirus associated with hydropericardium hepatitis syndrome in broiler and layer breeders in Azerbaijan

**DOI:** 10.1186/s12917-024-04081-0

**Published:** 2024-06-07

**Authors:** Erhan Bayraktar, Ozge Aydin, Hasan Emre Tali, Semaha Gul Yilmaz, Aysun Yilmaz, Nuri Turan, Ozge Erdogan Bamac, Akay Ozturk, Altug Erdem, Metin Kelleci, Jean-Remy Sadeyen, Pengxiang Chang, Huseyin Yilmaz, Munir Iqbal

**Affiliations:** 1 CEVA Animal Health, Poultry Division, Maslak, Türkiye; 2 Department of Virology, Istanbul University-Cerrahpasa, Veterinary Faculty, Hadimkoy, Istanbul, Türkiye; 3 The Pirbright Institute, Ash Road, Pirbright, Woking, GU24 0NF UK; 4 Department of Pathology, Istanbul University-Cerrahpasa, Veterinary Faculty, Hadimkoy, Istanbul, Türkiye; 5 Kartallar Veterinary Consultancy Company, Bursa, Türkiye

**Keywords:** Fowl adenovirus, Phylogenetic, Hydropericardium hepatitis syndrome, Gross lesions, Chickens, Azerbaijan

## Abstract

**Background:**

Fowl adenovirus-4 is a causative agent of hydropericardium hepatitis syndrome (HHS) in chickens and has been frequently reported from many countries. Fowl adenoviruses cause severe disease and mortality in broiler and layer breeders in Azerbaijan. Therefore, in this study, pathological lesions and the dissemination of fowl adenovirus-4 into the visceral organs of infected birds were investigated as well as molecular characterisation of detected strains. For this, liver, heart and spleen from 20 necropsied chickens originated from a broiler breeder flock and a layer breeder flock were embeded on the FTA cards and the samples were analysed for adenovirus-DNA by PCR and sequencing.

**Results:**

The findings of necropsy in both broiler and layer breeder chickens were similar, and the liver was severely effected showing hepatitis, and the heart with hydropericardium lesions. The kidneys were swollen with haemorrhages and small white foci on the surface of the spleens were noted. Intestinal congestion and ecchymotic hemorrhages were also observed in some birds. Fowl adenovirus-4-DNA was detected by PCR in all collected organs of 20 birds. The sequence analysis revealed that fowl adenovirus-4 present in Azerbaijan and close similarity of the *hexon* genes of the adenoviruses existing in the Middle East, North America, far east and Indian subcontinent were determined by phylogenetic analysis. However, sequence diversity was detected from the adenovirus strains circulating in Europe, North and South America.

**Conclusions:**

This study indicates the impact of fowl adenovirus-4 on the poultry health and production, and improved disease control and prevention strategies are necessary to reduce the HHS disease in chickens in Azerbaijan.

## Background

Avian adenoviruses cause variety of diseases in different bird species including chickens, ducks, quails, ostriches, falcons, raptors, psittacines and parrots [1]. Adenovirus infection (Inclusion body hepatitis) was first reported in broilers in the USA in 1963 [2]. Up to date, fowl adenoviruses (FAdVs) have been detected in chickens as causative agents of inclusion body hepatitis (IBH), hydropericardium hepatitis syndrome (HHS), adenoviral gizzard erosion (AGE), avian adenoviral splenomegaly (AAS) and egg drop syndrome (EDS) [3–5]. Recently, IBH and HHS have been frequently reported in commercial chickens from several countries causing significant economical losses [6–17].

FAdVs are non-enveloped double-stranded DNA viruses, which belong to the family *Adenoviridae,* composed of 720 hexons arranged in 240 trimers and 12 vertex pentons [1, 18–20]. Three main structural proteins of FAdVs capsids are hexon, fiber and penton base. The hexon gene is prone to mutations and used for serotyping as it harbors the major neutralizing epitopes [21]. The family adenoviridae contains six genera named as mastadenovirus, aviadenovirus, atadenovirus, siadenovirus and ichtadenovirus in addition to recently proposed testadenovirus of turtles and tortoises. Adenoviruses from three genera (aviadenovirus, siadenovirus, and atadenovirus) can infect birds [1, 5] FAdVs are classified into five different species (FAdV-A to FAdV-E) based on their molecular structure and also into 12 serotypes (FAdV-1-8a, 8b-11), as a result of cross-neutralization tests [1, 22]. At least 12 genotypes were identified within the five FAdV species based on the hexon gene sequences [21, 23]. FAdV-D (FAdV-2 and FAdV-11) and FAdV-E (FAdV-8a and FAdV-8b) commonly associated with IBH while HHS caused by FAdV-C (FAdV-4). FAdV-A (FAdV-1) has been isolated from most cases of gizzard erosion [1, 10, 22].

There are several methods to diagnose and identify FAdVs in chickens. Real time PCR for rapid diagnosis and PCR for sequencing the hexon gene which allows the differentiation of field isolates to species. In addition, serological tests like serum neutralisation are used to investigate the serotypes of FAdVs (Schachner et al., 2016). FAdVs are transmitted vertically and horizontally via all excretions, but the highest titers are found in feces and therefore fecal–oral transmission is very efficient way of transmission [19]. Because of rapid spread via feces and emergence of hypervirulent strain in China, outbreaks have been reported in the Middle east, Africa, Asia and recently in the USA [8–17]. However, there is no report about the occurrence of this disease complex in Azerbaijan at present. Also commercial vaccines are not being used against FAdVs in Azerbaijan due to lack of knowledge about disease frequency and circulating viruses. The aim of this study was to investigate outbreaks of adenoviral disease causing mortalities in broiler flocks to determine circulating strains and genetic diversity of FAdVs in Azerbaijan in relation to clinico-pathological signs.

## Results

### Clinical findings

The mortality up to 20% was the first prominent clinical findings in the suspected fowl adenovirus-4 infected chickens. There was a slight increase in mortality by the 7 and 9 weeks of age in both broiler and layer breeder flocks, respectively. In addition, lethargy, ruffled feathers, depression, decreased feed intake and egg production were also observed.

### Postmortem findings

**Fig. 1:**
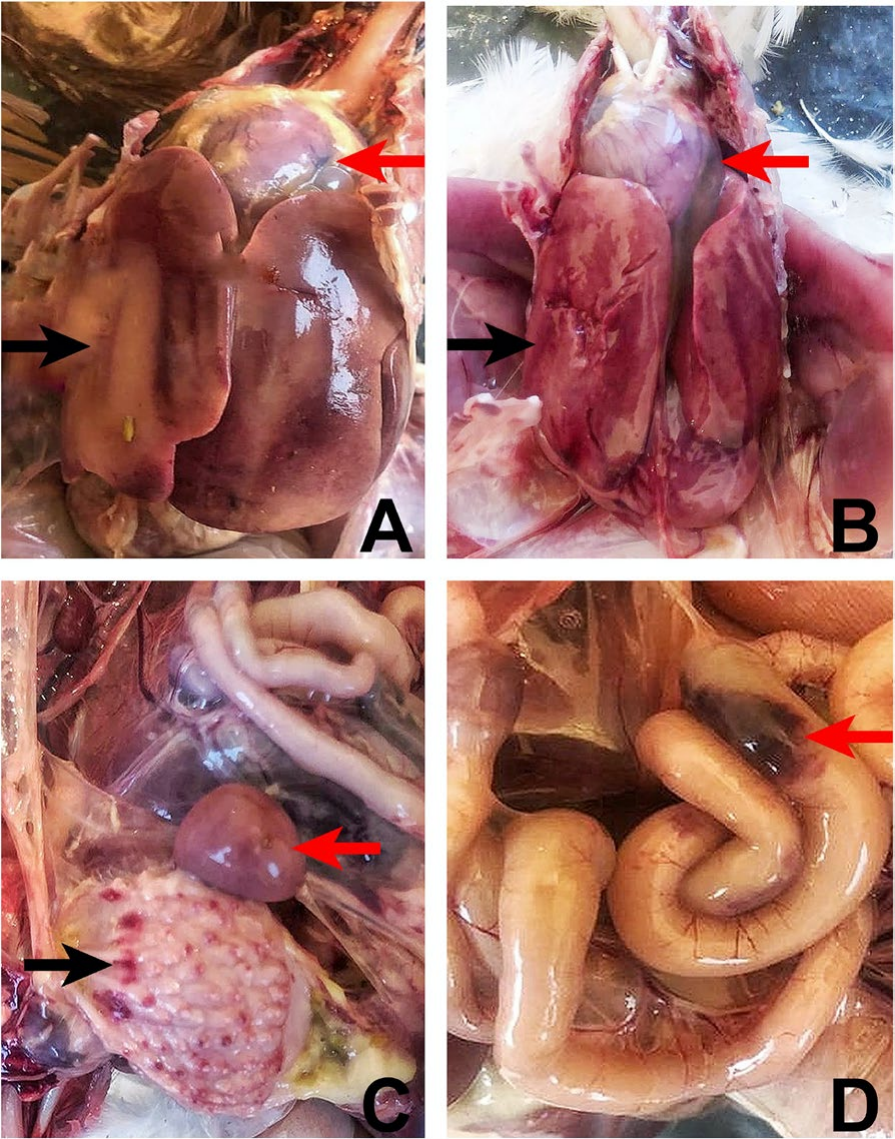
Gross lesions observed in the liver, heart, proventriculus, spleen and intestines of FAdV-4 positive chickens.** A**, **B** Swollen, friable liver with pale areas and hemorrhages (black arrows). Severe hydropericardium with an accumulation of clear, straw-colour, watery or jelly-like fluid in the pericardial sac (red arrows). **C** Petechial hemorrhages in the proventricular mucosa (black arrow). The spleen is prominent with white necrotic spots on its surface. **D** Congestion and echymotic hemorrhages in the intestines (red arrow)

Postmortem findings of both broiler and layer breeder flocks were similar and the most affected organs were the liver-hepatitis and the heart-hydropericardium. The livers were enlarged, friable, and pale with petechial and/or ecchymotic haemorrhages (Fig. 1-A and B). Although not observed in all the chickens which had hepatitis, hydropericardium was distinct with an accumulation of clear to straw-colored, watery or jelly-like fluid in the pericardial sac, giving the heart a misshapen and flabby appearance (Fig. 1-A and B). The kidneys were swollen and haemorrhagic in about 60% of birds.The spleens showed small white foci on the surface with splenomegaly signs. There were petechial hemorrhages in the mucosa of the proventriculus of around 20% percent of chickens including 2 infectious bursal disease virus-RNA (IBDV-RNA) positive animals (Fig. 1-C). Congestion and ecchymotic hemorrhages in the intestines were also remarkable (Fig. 1-D).

### PCR findings of other viral pathogens of chickens

When samples were screened for the presence of possible mixed viral infections by PCR, only IBDV-RNA was detected in 2 samples taken from the broiler breeders and layer breeders. The sequence and phylogenetic analysis of these viruses revealed that they were belong to very virulent strain of IBDV.

### Genotype findings of fowl adenoviruses

When DNA extracts subjected to PCR to amplify *hexon* genes of fowl adenovirus-4, a DNA band of PCR product 590 bp was observed on agarose gel in all collected organs of 20 birds. Sequence analysis and phylogenetic analyses were performed to detemine the phylographic realtionship of observedd sequences.

**Fig. 2:**
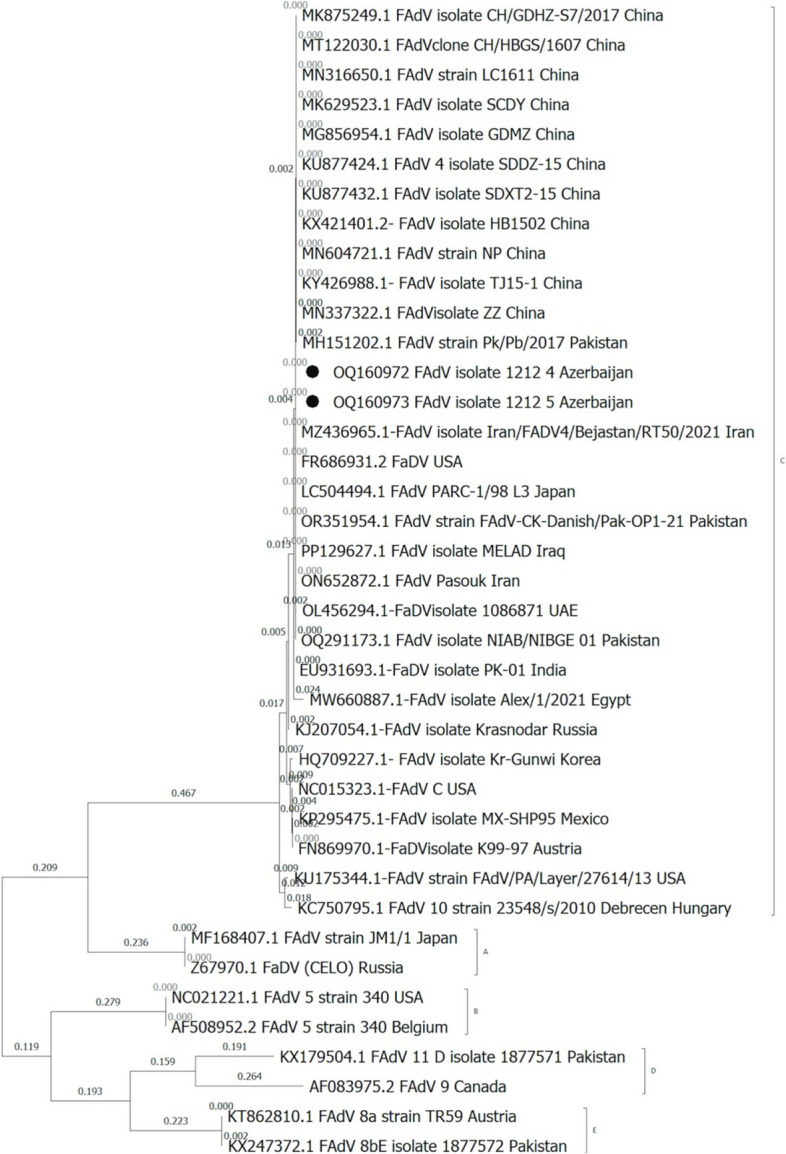
Phylogenetic tree of hexon genes of representative avian adenoviruses and FAdV-4 viruses. Gene sequences of the strains (OQ160972 and OQ160973-in black circles) detected in this study and representative adenovirus strains constructed by the maximum likelihood method in MEGA X. Bootstrap majority consensus values based on 1000 replicates are indicated at each branch point as a percentage

A phylogenetic tree, based on the sequences of the 507 bp *hexon* genes, generated five distinct clusters of fowl adenovirus namely A, B, C, D and E (Fig. 2). The FAdVs detected in this study were clustered in the species FAdV-C with 100% nucleodite sequence homology within the amplified hexon gene of fowl adenovirus-4 (Fig. 2). The sequences of FAdV-C obtained in this study shared 100% nucleotide identity to each other (broiler and layer breeders) and between 94.9% and 100% identity with the previously published sequences from other countries (BLAST, NCBI http://blast.ncbi.nlm.nih.gov/Blast.cgi.; Fig. 2 and Table 1). Since all the sequences were similar, only two sequences representing broiler and layer breeder flocks were submitted to GenBank (OQ160972 and OQ160973) and those sequences were used for phylogenetic analyses. All the sequences obtained from the liver and heart were also 100% identical.

**Table 1:**
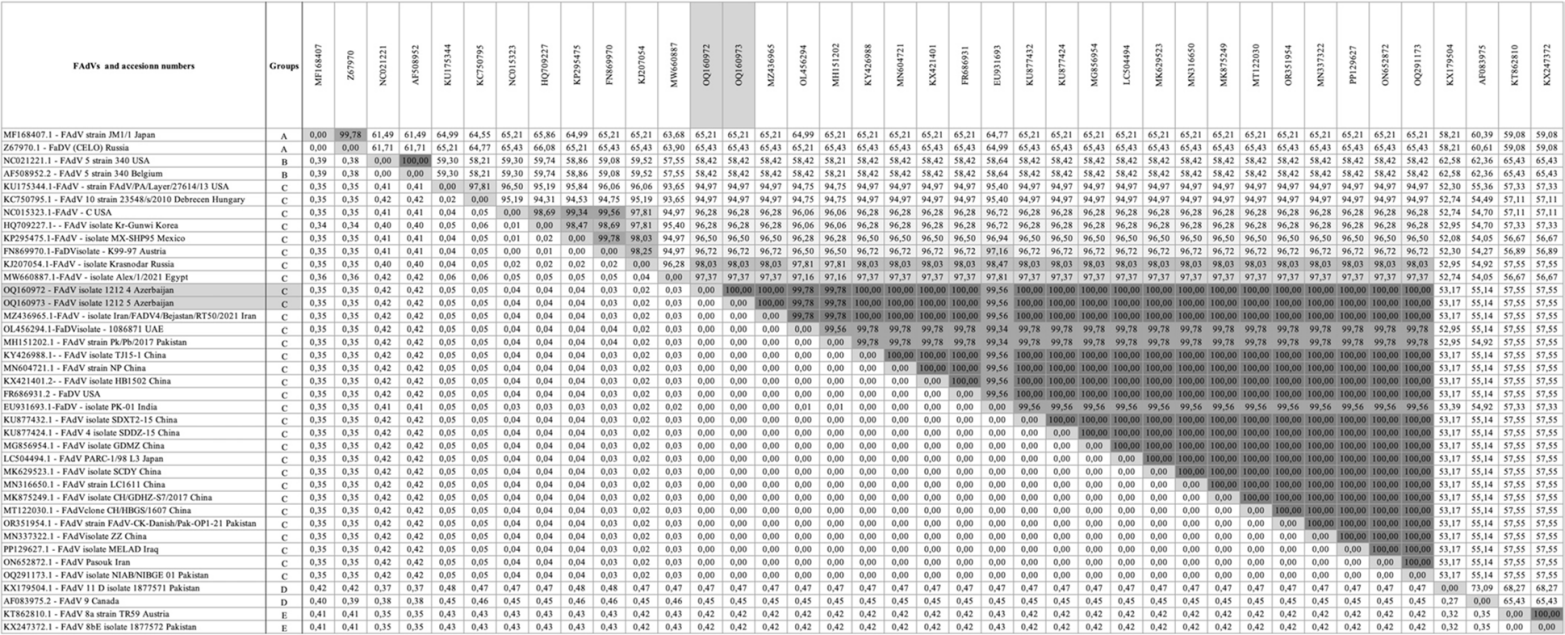
Nucleotide homologies and distances of the FAdV-4 strains detected in this study (OQ160972.1 and OQ160973.1) and strains detected in other countries

Results of the nucleotide percentage of homology and distance studies indicated that the strains detected in this study had 100% homologous identity with the FAdV-4 reported from Iran (MZ436965, ON652872), Iraq (PP129627), China (KU877424 and others), Pakistan (OR351954, OQ291173), USA (FR686931) and Japan (LC504494) (Table 1). The sequences also had 99,7% homology with FAdV-4 virulent strains reported from United Arab Emirates (OL456294), and from Pakistan (MH151202), 99.5% from India (EU931693), 98% from Russia (KJ207054), 97.3% from Egypt (MW660887), 96.7% from Austria (FN869970), 96.7 from Mexico (KP295475), 96.2% from USA (NC015323) and Korea (HQ709227), 94.9% from USA (KU175344) and Hungary (KC750795) (Table 1). The phylogenetic analysis of the hexon gene of FAdV-4 strains against the strains downloaded from NCBI of FAdV-4 in Fig. 2 revealed high proximity with FAdV-4 strains reported from Iran, United Arab Emirates, China, USA, Japan and Pakistan but showed diversity from FadV-4 strains of India, Russia, Egypt, Korea, USA, Austria, Mexico and Hungary (Fig. 2).

## Discussion

Hydropericardium hepatitis syndrome (HHS), formerly called hydropericardium syndrome (HPS) and Angara disease, was first described in 1987 in broiler chickens in Angara Goth, Pakistan [3]. Since 2015, after the detection of hypervirulent strain of FAdV-4 in China [24, 25] HHS cases have increased and severe outbreaks of HHS have been reported in chicken flocks in many countries like Iran, India, China, Egypt, United Arab Emirates, Poland and USA causing economical losses especially in 3–5 weeks old broilers with mortality rate up to 100% [6–17]. After the report of hypervirulent FAdV-4 strain and its rapid spread in China [24, 25] the emergent novel FAdV-4 serotype became very important virus to investigate in terms of molecular epidemiology and vaccine design. Since there is no report on the adenoviral diseases in chickens in Azerbaijan at present, outbreaks of HHS in broilers and layer breeders were investigated in this study to determine circulating strains and genetic diversity in relation to clinico-pathological signs.

Hexon and fiber proteins of FAdV are important structural proteins in virulence and host immune response to FAdVs and have been used for molecular characterisation by many investigators [7–17, 26]. In the present study, the hexon gene was used to investigate the phylogeny of the FAdVs. The phylogenetic analysis of hexon gene has shown that all the FAdVs detected in this study clustered within FAdV-C serotype FAdV-4. All the sequences were similar with 100% homology and therefore only two sequences to represent each broiler and layer breeder flock were submitted to GenBank. They also showed 100% homology with the strain detected in a neighbouring country Iran, and this might indicate that common ancestor virus is circulating because of the trade between these two countries. In addition, the phylogenetic analysis of the hexon gene of FAdV-4 strains have revealed that high proximity with FAdV-4 strains reported from Iran, Iraq, United Arab Emirates, China, Japan, USA, and Pakistan was found but sequences showed diversity from FadV-4 strains of India, Russia, Egypt, Korea, USA, Austria, Mexico and Hungary. These results suggest that strains detected in this study might have been deriven from a common ancestor FAdV-4 virus circulating amongst neighboring regions. Similarly, it is possible that strains detected in this study might have been originated from the virulent strain detected in China in 2015 [24, 25] since the 100% homology was observed with virulent strain of China [25] as well as the severity of clinical signs and pathological lesions observed in this study. Alternatively, the FAdV analyzed in this study, which shows high genetic similarity to strains endemic in countries like Iran, Iraq, United Arab Emeirates, China, Japan, USA, and Pakistan, may have been introduced through wild bird migration or the international trade of poultry products. However, the epidemiological relationship is unclear at present and warrants further investigations.

Although the HHS mostly reported from the broiler flocks after 2 weeks of hatch, breeding and laying flocks can also be affected with less frequency [7] as we have seen in this study. The mortality rates in broilers may reach up to 100% [16, 17]. However, mortality rate is lower when chickens get older since FAdV-4 infections are found to be age related [27]. Similar findings were found in this study. There is a clear age effect with avian adenoviruses, as the age of the host increases, the degree of multiplication of the viruses within the host is restricted and the mortality decreases. In a recent study, the pathogenicity of the hypervirulent (hvFAdV-4 strain GD616) in chickens were investigated and it was found that chickens younger than 59-day-old showed 100% morbidity and mortality, while 180-day-old chickens still exhibited a hydropericardium syndrome with 60% morbidity and 20% mortality [27]. Similarly, the highest mortality rate observed in this study was 20%. However, Chen and others [7] reported an HHS outbreak that occurred in a 100-day-old replacement pullet flock with 60% mortality.

The clinical symnptoms ruffled feathers, depression, dullness, varrying degrees of diarrhea, reduced feed intake, reduced performance and lack of uniformity seen in the present study were similar to those reported previously [15–17]. It has been well documented that hapatic lesions seen in IBH and HHS cases are similar but the only distinguishing feature between these two diseases is the presence of cardiac lesions and accumulation of fluid in the pericardial sac [28]. In the present stuy, lesions were detected in liver, heart, kidneys, spleen, proventriculus and intestine but the most affected organs were liver, heart and kidneys. The typical pathological findings at necrropsy like clear, straw-colored fluid accumulation in the pericardial sac, enlarged, friable and pale yellow liver with multiple haemorrhages, enlarged spleen with necrotic foci on their surface and swollen haemorrhagic kidneys were also similar to those reported previously [7, 11, 15–17, 28] However, petechial haemorrhages seen in the proventriculus have been reported in HHS cases although the adenoviral gizzard erosions (AGE) has been reported due to adenovirus serotype 1 (FAdV-1) infections in broiler chickens [29]. However, swelling of the proventriculus was seen in experiementally infected Specified pathogen free (SPF) chickens and proventricular bleeding observed in commercial chickens [30]. All of these findings indicate that FAdV-4 might be affecting glandular stomach [4, 30]. However, lesions seen in proventriculus is most lkely to be the consequence of very virulent IBDV infection detected in this study since it has been previously reported in vvIBDV infection [31].

There are some repeorts that co-infections with immunosuppressive chicken viruses like Marek’s disease virus (MDV), infectious bursal disease virus, (IBDV), chicken anemia virus (CAV), Avian metapneumovirus (AMPV) or infectious laryngotracheitis virus (ILTV), may exacerbate the FAdV pathogensis in chickens [6, 19, 32] For this reasion, we also analysed the presence of infectious bronchitis virus (IBV), MDV, IBDV aMPV, and ILTV in both flocks. Presence of vvIBDV infection was determined indicating that IBDV may have affected the disease severity. In conrast, there are reports showing the occurence of severe FAdV infections in the absence of immunosuppressive viruses [12, 18, 33].

## Conclusion

This is the first report detailing the genetic composition of FAdVs and the HHS disease with severe hepatitis and hydropericardium caused by FAdV-4 in broiler and layer breeders in Azerbaijan. The results provide an evidence that continued prevlence of virulent strains of FadVs in chickens flocks is becoming a serious concerns for the poultry production in the central Asian countries. Increased diseases burden along with severe economical loses requires an effective diseases control stratagies including availability of efficacious. The data on disease burden, epidemiological studies together from genotype to phenotype of prevaling fowl adenoviruses and their association with overall damage to poultry production in Azerbaijan is important in the development of frame work for implementation of disease preventative measures (diagnostics and vaccination). Our data will contribute in this effort for the development and implementation of appropriate effective vaccines to prevent and control the IBH and HHS diseases in chickens in Azerbaijan.

## Materials and methods

### Farms and study population

Mortality up to %20 were observed in a broiler breeder flock (Ross 308) and a layer breeder flock (Hyline-Sonja) in Azerbaijan in December 2022. Broiler breeder flock consisted of 40,000 birds and layer breeder flock 12,000 birds. The age of broiler breeder flock was 124 days and layer breeder flock 113 days. Hygienic conditions of the farms were good and biosecurity measurements were applied. According to flock records, birds were vaccinated with live Newcastle disease virus and IBV vaccines via spray as well as ILT vector MDV vaccines by the subcutaneous route in the hatchery and followed by Newcastle disease virus (NDV), IBV, IBDV, avian rhinotracheitis virus (ARTV) live vaccines via spray/drinking water and poxvirus vaccine via wing web, AEV via drinking water on farm. Flocks were not vaccinated with adenovirus vaccine.

### Necropsy

Necropsy of the 20 chickens (12 from broiler breeder and 8 from layer breeder) was performed on-site in sick birds by a consultant veterinarian. All dead animals were included in the study. Samples of the liver, heart and spleen taken from necropsied animals were embedded to FTA cards as described by the manufacturer (QIAcard™ Non-Indicating FTA™ Cards- WHAWB120210) and submitted to the Department of Virology of the Veterinary Faculty of Istanbul University-Cerrahpasa. The tests performed in this study were in the context of routine diagnosis and research activities, and no experimental studies were performed during the study. Therefore, no ethical issue is the concern of this study.

### Nucleic acid axtraction and reverse transcription for RNA viruses

Three punch samples (about 2 mm) were taken from each FTA card which have different tissues from different chickens. 200 μL of QIAcard FTA wash buffer (QIAcard FTA Wash Buffer (Cat. No. WB120112, Qiagen) were added on the samples and incubated for 5 min by vortexing. 100 μL of nuclease-free water was added to each sample. Viral DNA and RNA were extracted from these suspensions to detect FAdVs by using a commercial DNA/RNA extraction kit (innuPREP virus DNA/RNA kit, 854 KS, IST Innuscreen) according to the manufacturer’s protocol (IST Innuscreen, Germany). DNA/RNA was eluted in 30 μL of elution buffer and stored at − 20 °C until used. Reverse transcription was performed by using a high-capacity cDNA Reverse Transcription Kit (Applied Biosystems, Waltham, MA, USA) following the manufacturer’s instructions.

### PCR amplification of the Hexon Loop-1 region of Fowl adenovirus

The hexon gene was partially amplified by using PCR to confirm the presence of adenoviral DNA and for sequencing as described previously [10]. One set of primers binding to the hex loop 1 (L1) gene was used to amplify L1. The primers were as follows: Hex L1-F 5′-ATGGGAGCSACCTAYTTCGACAT-3′ (301–323) as the forward primer and Hex L1-R 5′-AAATTGTCCCKRAANCCGATGTA-3′ (890–868) as the reverse primer [10, 21, 34] Briefly, in an optimised PCR reaction, a total volume of 25 μL of reaction mixture containing 2 μL (10 μM) of each forward and reverse primer, 12.5 μL of Maxima Hot Start PCR Master Mix (Thermo Scientific, Waltham, MA, USA), 4.5 μL of nuclease-free water, 2 μL of MgCl2, and 2 μL of DNA were used to amplify 590 base pair (bp) of hexon gene under the protocol described previously [10, 21]. In all PCR reactions, positive and negative controls were included. A known positive field sample was used as the positive control, while nuclease-free water was included as the negative control in place of the DNA template. Following 1.5% agarose gel electrophoresis, amplified PCR products from the liver samples were sent for sequencing to a commercial company (MedSanTek, Turkey).

Samples were also analysed by PCR for the presence of IBV, IBDV, aMPV and MDV as described previously [35–38] and infectious laryngotracheitis virus (ILTV) by using *in house* method as part of routine diagnostic work.

### Sequencing and phylogenetic analysis

Nucleotide sequences of the partial hexon genes (590 bp) of FAdV were edited by Chromas Pro and aligned using the MAFFT version 7 (online version) [39]. To compare the genotypic relationship between FAdV strains of this study and other FAdV strains detected in other countries, multiple alignments of partial hexon gene sequences of the FAdV data available in the National Centre for Biotechnology Information were made using the MEGA-X software [40] Phylogenetic tree was generated by using Maximum Likelihood method and Hasegawa-Kishino-Yano (HKY) model with 1000 Bootstrap replicates by using the MEGA-X [40] Comparative percentage of homology and distance were determined by using DNASTAR software (MegAlignPro, Version 17.5.0–2023). Two FAdV field strains (representative of broiler and layer breeder flocks) detected in this study (in Azerbaijan) were submitted to GenBank under the submission numbers (OQ160972 and OQ160973).

## Data Availability

The data of this study are included in the manuscript. The data is available up on request from the corresponding author. Accession numbers of two FAdV field strains submitted to GenBank are OQ160972 and OQ160973.

## Abbreviations

AGE: Adenoviral gizzard erosion
AAS: Avian adenoviral splenomegaly
EDS: Egg drop syndrome
FadV: Fowl adenovirus
HHS: Hydropericardium hepatitis syndrome

## References

[1] Hess M, Swayne DE, Bouliann M, Logue CM, McDougald LR, Nair V, Suarez DL, Wit S de, Grimes T, Johnson D, Kromm M, Prajitno TY, Rubinoff I, Zavala G (2020). Aviadenovirus infections. Diseases of poultry.

[2] Helmboldt CF, Frazier M (1963). N Avian hepatic inclusion bodies of unknown significance. Avian Dis.

[3] Anjum AD, Sabri MA, Iqbal Z (1989). Hydropericarditis syndrome in broiler chickens in Pakistan. Vet Rec.

[4] Li PH, Zheng PP, Zhang TF, Wen GY, Shao HB, Luo QP (2017). Fowl adenovirus serotype 4: Epidemiology, pathogenesis, diagnostic detection, and vaccine strategies. Poult Sci.

[5] El-Shall NA, El-Hamid HSA, Elkady MF, Ellakany HF, Elbestawy AR, Gado AR, Geneedy AM, Hasan ME, Jaremko M, Selim S, El-Tarabily KA, El-Hack MEA (2022). Corrigendum: Epidemiology, pathology, prevention, and control strategies of inclusion body hepatitis and hepatitis-hydropericardium syndrome in poultry: A comprehensive review. Front Vet Sci.

[6] Niczyporuk JS (2016). Phylogenetic and geographic analysis of fowl adenovirus field strains isolated from poultry in Poland. Arch Virol.

[7] Chen L, Yin L, Zhou Q (2019). Epidemiological investigation of fowl adenovirus infections in poultry in China during 2015–2018. BMC Vet Res.

[8] Wang K, Sun H, Li Y (2019). Characterization and pathogenicity of fowl adenovirus serotype 4 isolated from eastern China. BMC Vet Res.

[9] Wibowo MH, Sahesty A, Mahardika BK, Purwanto B, Lestariningsih CL, Kade Suardana IB, Oka Winaya IB, Irine I, Suryanggono J, Jonas M (2019). Epizootiology, Clinical Signs, and Phylogenetic Analysis of Fowl Adenovirus in Chicken Farms in Indonesia from 2018 to 2019. Avian Dis.

[10] Raue R, Gerlach H, Müller H (2005). Phylogenetic analysis of the hexon loop 1 region of an adenovirus from psittacine birds supports the existence of a new psittacine adenovirus (PsAdV). Arch Virol.

[11] Yuming F, Sheng Y, Wenyu D, Shihong C, Wenfeng L, Wenjing H, Xiaowen L, El-Ashram S, Mei K, Jinyue G (2020). Molecular characterization and phylogenetic analysis of fowl adenovirus serotype-4 from Guangdong Province. China Vet World.

[12] Chitradevi S, Sukumar K, Suresh P, Balasubramaniam GA, Kannan D (2021). Molecular typing and pathogenicity assessment of fowl adenovirus associated with inclusion body hepatitis in chicken from India. Trop Anim Health Prod.

[13] Lai VD, Min K, Lai HTL, Mo J (2021). Epidemiology of fowl adenovirus (FAdV) infections in South Korean chickens during 2013–2019 following introduction of FAdV-4 vaccines. Avian Pathol.

[14] Mete A, Armien AG, Rejmanek D, Mott M, Crossley BM (2021). Emergence of fowl aviadenovirus C-4 in a backyard chicken flock in California. J Vet Diagn Invest.

[15] Sultan H, Arafa AE, Adel A, Selim K, Hossiny M, Talaat S (2021). Molecular Detection of a Novel Fowl Adenovirus Serotype-4 (FadV-4) from an Outbreak of Hepatitis Hydropericardium Syndrome in Commercial Broiler Chickens in Egypt. Avian Dis.

[16] Ishag HZA, Terab AMA, El Tigani-Asil ETA, Bensalah OK, Khalil NAH, Khalafalla AI, Al Hammadi ZMAH, Shah AAM, Al Muhairi SSM (2022). Pathology and Molecular Epidemiology of Fowl Adenovirus Serotype 4 Outbreaks in Broiler Chicken in Abu Dhabi Emirate, UAE. Veterinary Sciences..

[17] Toroghi R, Sodavari S, Tabatabaeizadeh SE, Sharghi AS, Irankhah N, Fakhraee M, Farzin HR, Sarani M, Khayyat SH, Ashouri M (2022). The First Occurrence of Hepatitis-Hydropericardium Syndrome in Iran and Effective Applied Control Measures in the Affected Commercial Broiler Flock. Avian Dis.

[18] Steer PA, Kirkpatrick NC, O'Rourke D, Noormohammadi AH (2009). Classification of fowl adenovirus serotypes by use of high-resolution melting-curve analysis of the hexon gene region. J Clin Microbiol.

[19] Schachner A, Matos M, Grafl B, Hess M (2018). Fowl adenovirus-induced diseases and strategies for their control - a review on the current global situation. Avian Pathol.

[20] Benkő M, Aoki K, Arnberg N, Davison AJ, Echavarría M, Hess M, Jones MS, Kaján GL, Kajon AE, Mittal SK (2022). ICTV Virus Taxonomy Profile: *Adenoviridae* 2022. J Gen Virol.

[21] Schachner A, Marek A, Grafl B, Hess M (2016). Detailed molecular analyses of the hexon loop-1 and fibers of fowl aviadenoviruses reveal new insights into the antigenic relationship and confirm that specific genotypes are involved in field outbreaks of inclusion body hepatitis. Vet Microbiol.

[22] Schachner A, Hess M (2022). Special Issue: Avian Adenoviruses Viruses.

[23] Marek A, Schulz E, Hess C, Hess M (2010). Comparison of the fibers of Fowl adenovirus A serotype 1 isolates from chickens with gizzard erosions in Europe and apathogenic reference strains. J Vet Diagn Invest.

[24] Ye J, Liang G, Zhang J, Wang W, Song N, Wang P, Zheng W, Xie Q, Shao H, Wan Z (2016). Outbreaks of serotype 4 fowl adenovirus with novel genotype, China. Emerg Microbes Infect.

[25] Liu A, Zhang Y, Cui H, Wang X, Gao Y, Pan Q (2022). Advances in Vaccine Development of the Emerging Novel Genotype Fowl Adenovirus 4. Front Immunol.

[26] Zhang Y, Liu R, Tian K, Wang Z, Yang X, Gao D, Zhang Y, Fu J, Wang H, Zhao J (2018). Fiber2 and hexon genes are closely associated with the virulence of the emerging and highly pathogenic fowl adenovirus 4. Emerg Microbes Infect.

[27] Yuan F, Song H, Hou L, Wei L, Zhu S, Quan R, Wang J, Wang D, Jiang H, Liu H (2021). Age-dependence of hypervirulent fowl adenovirus type 4 pathogenicity in specific-pathogen-free chickens. Poult Sci.

[28] Niu YJ, Sun W, Zhang GH, Qu YJ, Wang PF, Sun HL, Xiao YH, Liu SD (2016). Hydropericardium syndrome outbreak caused by fowl adenovirus serotype 4 in China in 2015. J Gen Virol.

[29] Mirzazadeh A, Grafl B, Abbasnia M, Emadi-Jamali S, Abdi-Hachesoo B, Schachner A, Hess M (2021). Reduced Performance Due to Adenoviral Gizzard Erosion in 16-Day-Old Commercial Broiler Chickens in Iran. Confirmed Experimentally Front Vet Sci.

[30] Jiang Z, Liu M, Wang C, Zhou X, Li F, Song J, Pu J, Sun Y, Wang M, Shahid M (2019). Characterization of fowl adenovirus serotype 4 circulating in chickens in China. Vet Microbiol.

[31] Islam MT, Samad MA (2004). Clinico-pathological studies on natural and experimental infectious bursal dis- ease in broiler chickens. Bangladesh J Vet Med.

[32] Choi KS, Kye SJ, Kim JY, Jeon WJ, Lee EK, Park KY, Sung HW (2012). Epidemiological investigation of outbreaks of fowl adenovirus infection in commercial chickens in Korea. Poult Sci.

[33] Gomis S, Goodhope AR, Ojkic AD, Willson P (2006). Inclusion body hepatitis as a primary disease in broilers in Saskatchewan. Canada Avian Dis.

[34] Pilkington P, Brown T, Villegas P, McMurray B, Page RK, Rowland GN, Thayer SG (1997). Adenovirus-Induced Inclusion Body Hepatitis in Four-Day-Old Broiler Breeders. Avian Dis.

[35] Yilmaz H, Altan E, Cizmecigil UY, Gurel A, Ozturk GY, Bamac OE, Aydin O, Britton P, Monne I, Cetinkaya B (2016). Phylogeny and S1 Gene Variation of Infectious Bronchitis Virus Detected in Broilers and Layers in Turkey. Avian Dis.

[36] Bayraktar E, Umar S, Yilmaz A, Turan N, Franzo G, Tucciarone CM, Cecchinato M, Cakan B, Iqbal M, Yilmaz H (2018). First Molecular Characterization of Avian Metapneumovirus (aMPV) in Turkish Broiler Flocks. Avian Dis.

[37] Yilmaz A, Turan N, Bayraktar E, Gurel A, Cizmecigil UY, Aydin O, Bamac OE, Cecchinato M, Franzo G, Tali HE (2019). Phylogeny and evolution of infectious bursal disease virus circulating in Turkish broiler flocks. Poult Sci.

[38] Yilmaz A, Turan N, Bayraktar E, Tali HE, Aydin O, Umar S, Cakan B, Sadeyen JR, Baigent S, Iqbal M (2020). Molecular characterisation and phylogenetic analysis of Marek's disease virus in Turkish layer chickens. Br Poult Sci.

[39] Kumar S, Stecher G, Tamura K (2016). MEGA7: molecular evolutionary genetics analysis version 7.0 for bigger datasets. Mol. Biol. Evol..

[40] Kumar S, Stecher G, Li M, Knyaz C, Tamura K (2018). MEGA X: Molecular evolutionary genetics analysis across computing platforms. Mol Biol Evol.

